# Using direct observations on multiple occasions to measure household food availability among low-income *Mexicano *residents in Texas *colonias*

**DOI:** 10.1186/1471-2458-10-445

**Published:** 2010-07-29

**Authors:** Joseph R Sharkey, Wesley R Dean, Julie A St John, J Charles Huber

**Affiliations:** 1 Program for Research in Nutrition and Health Disparities, School of Rural Public Health, Texas A&M Health Science Center, MS 1266, College Station, TX, USA; 2 Department of Social and Behavioral Health, School of Rural Public Health, Texas A&M Health Science Center, College Station, TX, USA; 3 Center for Community Health Development, School of Rural Public Health, Texas A&M Health Science Center, College Station, TX, USA; 4 Comidas Saludables & Gente Sana en el Sur de Tejas (Healthy Food and Healthy People in South Texas), School of Rural Public Health, Texas A&M Health Science Center, College Station, TX, USA; 5 Department of Epidemiology and Biostatistics, School of Rural Public Health, Texas A&M Health Science Center, College Station, TX, USA

## Abstract

**Background:**

It has been recognized that the availability of foods in the home are important to nutritional health, and may influence the dietary behavior of children, adolescents, and adults. It is therefore important to understand food choices in the context of the household setting. Considering their importance, the measurement of household food resources becomes critical.

Because most studies use a single point of data collection to determine the types of foods that are present in the home, which can miss the change in availability within a month and when resources are not available, the primary objective of this pilot study was to examine the feasibility and value of conducting weekly in-home assessments of household food resources over the course of one month among low-income *Mexicano *families in Texas *colonias*.

**Methods:**

We conducted five in-home household food inventories over a thirty-day period in a small convenience sample; determined the frequency that food items were present in the participating households; and compared a one-time measurement with multiple measurements.

After the development and pre-testing of the 252-item culturally and linguistically- appropriate household food inventory instrument that used direct observation to determine the presence and amount of food and beverage items in the home (refrigerator, freezer, pantry, elsewhere), two trained *promotoras *recruited a convenience sample of 6 households; administered a baseline questionnaire (personal info, shopping habits, and food security); conducted 5 in-home assessments (7-day interval) over a 30-day period; and documented grocery shopping and other food-related activities within the previous week of each in-home assessment. All data were collected in Spanish. Descriptive statistics were calculated for mean and frequency of sample characteristics, food-related activities, food security, and the presence of individual food items. Due to the small sample size of the pilot data, the Friedman Test and Kendall's W were used to assess the consistency of household food supplies across multiple observations.

**Results:**

Complete data were collected from all 6 *Mexicano *women (33.2y ± 3.3; 6.5 ± 1.5 adults/children in household (HH); 5 HH received weekly income; and all were food insecure. All households purchased groceries within a week of at least four of the five assessments. The weekly presence and amounts of fresh and processed fruits and vegetables, dairy, meats, breads, cereals, beverages, and oils and fats varied. Further, the results revealed the inadequacy of a one-time measurement of household food resources, compared with multiple measures. The first household food inventory as a one-time measure would have mistakenly identified at least one-half of the participant households without fresh fruit, canned vegetables, dairy, protein foods, grains, chips, and sugar-sweetened beverages.

**Conclusions:**

This study highlights the value of documenting weekly household food supplies, especially in households where income resources may be more volatile. Clearly, the data show that a single HFI may miss the changes in availability - presence and amount - that occur among low-income *Mexicano *households who face challenges that require frequent purchase of foods and beverages. Use of multiple household food inventories can inform the development and implementation of nutrition-related policies and culturally sensitive nutrition education programs.

## Background

The burden of obesity and nutrition-related health conditions, such as type 2 diabetes, hypertension, and coronary heart disease, disproportionately affects children and marginalized populations that face increasing vulnerability to food insecurity and poor nutrition health [1,2]. One such marginalized population is *Mexicano *(Mexican-origin) families who reside in impoverished *colonias *along the Texas-Mexico border [3]. *Colonias*, developed from subdivided agricultural lands in response to a deficit in low-income housing [4], are substandard residential areas often with inadequate roads, variable housing conditions, and limited access to safe water and sewer sources [5]. Almost 20% of these largely *Mexicano *households have a female head, and 50% of children are food insecure [6,7]. Notably, the border population is growing at a rate nearly double that of the rest of Texas [8]. Obesity and related health conditions predominate among Mexican Americans in the Lower Rio Grande Valley (LRGV) of Texas. Of great concern are increases in overweight and obesity among Mexican American children in the LRGV [9]. The term *Mexicano *is being broadly used to refer to *colonia *residents who label themselves as Mexican in origin, regardless of birthplace [10].

Although obesity has risen at alarming rates among all segments of the population, prevalence is significant among Mexican Americans and continues to increase among the poor and near-poor [11-13]. The dramatic increase in obesity levels among the U.S. population is primarily due to an energy imbalance [14]. Energy-dense and nutrient-poor foods, such as sugar-sweetened beverages, high-fat baked goods, desserts, and salty/high-fat snacks, are highly palatable and promote higher calorie intakes [15,16]. For poor populations, energy-dense foods may also be more affordable and more accessible [16,17]. Considering the multifactorial nature of obesity and the increased risk for adverse health conditions faced by children who experience accelerated weight gain [2,16], understanding the factors that influence individual food choice and healthful eating is critical to addressing the obesity problem through nutrition education and policy. An ecological paradigm suggests that multiple levels of the food environment influence individual at-home dietary intake [18,19]. In particular, community and neighborhood retail food environments influence the household food environment through acquisition of food items, which may dictate food preparation and consumption choices made at home [20,21]. Access to community and neighborhood food resources may exert a greater influence for limited resource families [3,22]. As French and colleagues point out, the household food environment is an intermediate level between the retail food environment and individual dietary intake [20]. Despite research that has found that the accessibility to foods in the home is one of the most important determinants of eating behavior [16,23], the preponderance of published studies focus on access to the retail food environment [3,24-36].

National data suggest that home-prepared food accounts for approximately 61% of food expenditures and 68% of total calories [37,38]. For some subpopulations, such as *Mexicano *families along the Texas border with Mexico, the proportion of meals and calories consumed outside the home is relatively small [39]. Research has shown that the availability of foods in the home are important to nutritional health [22,40-44], and may indicate dietary behavior of children, adolescents, and adults [16,22,42,45-49]. Therefore, understanding food choices in the context of the household setting is important [20]. Moreover, considering the importance of household food resources, the measurement of household food resources becomes critical [20].

Two main approaches have been used to measure household food availability; one that documents food items coming into the home, using grocery receipts and records [20,50-53], and another that inventories actual food items present in the home, relying on mailed, telephone, or researcher-administered surveys and participant self-report [22,42,43,54-59] or direct observation by a trained researcher [60,61]. With limited-item or comprehensive household food inventories (HFIs), most researchers document household availability at a single point in time [20,22,40,43,54,57,62,63]. However, a single HFI is unable to address intra-household variation during the month in food resources, which may be influenced by weekly or bi-weekly shopping trips, economics (e.g., income cycles), demands of work outside the home, household refrigeration and storage, family events such as celebrations or family traumas and emotional events, bouts of acute illness, transformations in domicile, and food store access [64-66]. The intra-month variation in household availability may be a key factor in food intake. As French and colleagues pointed out, a single HFI does not capture variation in household food availability over time, much like a single dietary recall would not capture variations in dietary habits [20,67]. Another shortcoming in evaluating household food resources is the lack of consideration for the amounts of food and beverages present in the home, given the adequacy of food resources may relate to household size and composition [20,65,68,69].

Understanding intra-month variation in household food availability will better inform the development and implementation of nutrition education and nutrition-related policies. Most studies use a single point of data collection to determine the types of foods that are present in the home. Because this approach can miss the change in availability within a month and when resources are not available, the primary objective of this pilot study was to examine the feasibility and value of conducting weekly in-home assessments of household food resources over the course of one month among low-income *Mexicano *families in Texas *colonias*. Specifically, we conducted five in-home household food inventories over a thirty-day period in a small convenience sample; determined the frequency that food items were present in the participating households; and compared a one-time measurement with multiple measurements.

## Methods

### Setting and Participants

Since this was a pilot project and little data existed on feasibility of conducting five in-home food inventories during a 30-day period, the decision was made *a priori *to recruit and retain three participants from each of two geographic areas of *colonias *along the South Texas border with Mexico. Recruitment of six participants and data collection were conducted by local *promotoras *(indigenous community health workers) who were affiliated with the South Texas Center for Community Health Development; worked in two targeted areas of *colonias *in the eastern and western parts of Hidalgo County, which are approximately 20 miles apart; and have been involved in previous nutrition-related research projects. The *promotoras *contacted women with at least one child under the age of eighteen living in the household and who had participated in a prior research project. Three women were contacted and were recruited from *colonias *in the eastern part of Hidalgo County and five were contacted and three recruited from *colonias *in the western part of the county. All six women who agreed to participate in the study completed all five in-home assessments. The study was completed in July-September, 2008. Participants received a cash incentive for participation in the study, which was distributed at the end of the study. Informed consent was obtained from all participants, and the study was approved by the Institutional Review Board at Texas A&M University. All materials were translated into Spanish; reviewed for semantic and conceptual equivalence by the *promotoras*; and necessary modifications were made.

### Baseline Questionnaire

A questionnaire during the first in-home visit was administered by a *promotora*-interviewer in Spanish; and included the following sections: 1) sociodemographic characteristics, 2) food-related activities, and 3) food security. Once translated into Spanish, all questionnaire items were examined by project *promotoras *for conceptual and semantic equivalence; and appropriate changes were made. **Sociodemographic characteristics **included participant's age, education (highest grade completed in school), race/ethnicity, marital status, number of adults and children residing in the household, ages of children, household income in 2007, frequency of income payments, employment status of household members, automobile ownership, other sources of transportation, nutrition program participation (e.g., Supplemental Nutrition Assistance Program [SNAP], Women, Infants, and Children Nutrition Program [WIC], School Breakfast Program, and School Lunch Program), and health conditions among household members (e.g., diabetes, obesity, and heart problems). SNAP participants were also asked the length of time in the program and amount and receiving date of current benefits. **Food-related activities **included questions concerning the store where most of household's groceries were purchased; one-way distance and time to travel to that store; typical method of transportation; frequency of shopping at this store (weekly, bi-weekly, monthly, or less than once a month); amount spent on groceries; days since last food shopping occasion; and frequency of prepared meals purchased from a fast- or full-service restaurant for consumption at home or at the restaurant. **Food security **was measured using the U.S. Household Food Security Survey Module: Six-Item Short Form [70,71]. Food security status was determined from the occurrence of the following food security risk situations during the 12 months prior to the first home visit: purchased food did not last and money was not available to get more (often true, sometimes true, or never true); could not afford to eat balanced meals (often true, sometimes true, or never true); adults in the household cut the size of meals or skipped meals because there wasn't enough money for food (yes or no); adults eat less than they felt they should eat because there wasn't enough money for food (yes or no); and were hungry and did not eat because they couldn't afford enough food (yes or no). If the participant answered often or sometimes, a follow-up question asked whether or not this happened almost every month, only 1 or 2 months, or some months but not every month. Scores were calculated to classify households as food secure (score = 0), marginally food secure (score = 1), food insecure (score = 2-4), and high food insecurity (score = 5-6), which is similar to food insecure with hunger.

### Household Food Inventory (HFI)

The HFI instrument included 252 items and was modified from a 171-item shelf inventory survey used in low-income Latino families and a 251-item inventory used in rural Texas [59,65]. Food items were added to include canned and frozen fruit and vegetables and regional and cultural food items that were not included in the original version. The HFI consisted of the following categories: *verdura fresco *(fresh vegetables); *fruta fresco *(fresh fruit); *cereales *(cereals), *pan, pastels, galletas saladas y galletas *(bread, cakes, crackers, and cookies); *tortillas, pasta y arroz *(tortillas, pasta and rice); *leche, lecheria, helado, yogur y queso *(milk, dairy, ice cream, yogurt, and cheese); *carne, carne de aves, jamon y salchichas, frescas o congeladas *(meat, poultry, ham and sausage, fresh or frozen); *mariscos, frescos, congelados o enlatados *(fish, fresh, frozen or canned); *palomitas de maiz o papitas *(chips and popcorn); *legumbres *(legumes); *verduras - enlatadas/frasco *(vegetables - canned/jar); *frutas enlatadas *(canned fruit); *sopas y consomes *(broth and soups); *bebidas *(beverages); *articulos de comida micelanea *(miscellaneous pantry items); *verduras congeladas *(frozen vegetables); *frutas congeladas *(frozen fruit); *mayonesa, salsa y adereso *(mayonnaise, sauce, and salad dressing); *aceites y otras mantecas *(oils and other fats); and *comida para bebe *(baby food). Canned fruit was identified as being packed in *sirope espeso *(heavy syrup) or *sirope/jugo ligero *(light syrup or juice). The instrument was designed to document *si *(presence) and *cantidad *(amount) of each food item. Amounts were determined by a count of the number of items of whole fresh fruit and vegetables, labeling of bottled, canned, or prepackaged foods, and estimation of previously opened or sliced food items.

### Follow-up Questionnaire

A follow-up questionnaire was administered during home visits 2, 3, 4, and 5 to identify food-related activities that occurred since the prior home visit. The following questions were included: 1) were groceries purchased (where, how much was spent, type of purchase, and method of transportation); 2) did you/you and your family eat at a fast food restaurant (and frequency); 3) did you/you and your family eat a restaurant (and frequency); and 4) did you/you and your family purchase food prepared elsewhere to eat at home (and frequency). Frequency responses included once, 2-3 times, 4-5 times, >5 times, or does not apply.

### Data Collection

The *promotoras *completed a one-day training session, preview and testing of all materials, and follow-up training sessions. The training sessions included information on research with human subjects, maintaining confidentiality, and practice sessions in administering questionnaires and documenting the presence and amount of foods present during household food inventories. Data were collected in each participant's home by a team of two trained *promotoras *during five home visits, which were scheduled to occur over thirty days; each home visit was scheduled to occur approximately 6-7 days after the prior home visit. The study was conducted during the months of July, August, and early September 2008. During the first visit to each household, the baseline questionnaire was administered; the first HFI was completed using direct observation of food stored in the home; and photographs were taken of foods and food storage. During home visits 2-5, a follow-up questionnaire was administered; household food inventory was assessed; and photographs were taken. The *promotoras *were all fluent Spanish speakers from the local community who shared a common cultural background with the participants in the study.

### Data Analysis

Survey and household food inventory data were entered into a relational database (Microsoft Office Access 2007); descriptive statistics were calculated for mean and frequency of sample characteristics, food-related activities, food security, and the presence of individual food items, using Stata statistical software release 11.0 (Stata Corp., College Station, TX). Due to the small sample size of the pilot data, traditional multilevel modeling techniques which rely on large sample theory for accurate p-values were not appropriate. The Friedman Test and Kendall's W Test [72,73], two non-parametric techniques, were used to assess the consistency of household food supplies across multiple observations. The null hypothesis for both of these tests is that the contents of each participant's household are the same each time they are observed; that is, that the food or beverage item is present, regardless of quantity. Note that the tests do not differentiate between the presence or absence of a particular item - only that the contents are the same across all observations. While the Friedman and Kendall tests yield the same p-value, the Kendall W statistic has the advantage of being interpreted as a measure of concordance or agreement ranging from zero to one. A value of zero indicates no consistency across observations and a value of one indicates perfect consistency across observations. Both tests were implemented in Stata 11.0 using the "friedman" command written by Goldstein [73].

## Results

The sociodemographic characteristics of the six participant mothers and households are shown in Table 1. All participants considered themselves *Mexicano *and all data were collected in Spanish. Household size ranged from 4-8 adults and children; household composition included 2-4 adults and 2-6 children. All participants reported a household income ≤$15,000/yr, with a household income <$10,000/yr for five of the six participants. For most, income was received on no more than a weekly basis. Participants traveled 3-10 miles one-way to purchase groceries; half of them traveled with a friend or neighbor; and five of the six households spent <$400/month on groceries (data not shown). Groceries for the household were primarily purchased weekly or bi-weekly. The lack of food security is a problem for the participant households (see Table 2). Five of the six households evidenced high food insecurity (food insecurity with hunger), with the sixth household being food insecure.

**Table 1:** Sociodemographic Characteristics for all Six Participant Mothers

	Mean ± SD (range)	% (n)
Age (years)	33.2 ± 3.3 (27-36)	
Education (years completed)	8.2 ± 2.8 (6-13)	
Race/ethnicity		
Hispanic		100 (6)
Marital status		
Married		100 (6)
Household composition (number)		
Adults	2.3 ± 0.8 (2-4)	
Children	4.2 ± 1.3 (2-6)	
Total adults and children	6.5 ± 1.5 (4-8)	
Age of children in household (years)		
Household average	5.9 ± 4.8 (1-12)	
Household income (in thousands)/y		
<$10		83.3 (5)
$10-$15		16.7 (1)
Frequency of income		
Daily		16.7 (1)
Weekly		66.7 (4)
Bi-weekly		16.7 (1)
Household adults employed		
Participant (female)		0 (0)
Spouse		100 (6)
Transportation		
Car ownership		66.7 (4)
Neighbor^a^		33.3 (2)
Nutrition program participation		
Supplemental Nutrition Assistance Program (SNAP)		83.3 (5)
Women, Infants, and Children (WIC) Program		50 (3)
Free school breakfast		33.3 (2)
Free or reduced school lunch		0 (0)

^a ^Ride with neighbor (100% charge for transportation)

**Table 2:** Food Security Using the Six-Item Short Form of the Food Security Survey Module

	% (n)
In the past 12 months	
Food that was purchased did not last and didn't have money to get more	100 (6)
Almost every month^a^	33.3 (2)
Some months^a^	66.7 (4)
Could not afford to eat balanced meals	100 (6)
Almost every month^b^	33.3 (2)
Some months^b^	50 (3)
1-2 months^b^	16.7 (1)
Cut the size or skipped meals because there wasn't enough money for food	83.3 (5)
Almost every month^c^	16.7 (1)
Some months^c^	66.7 (4)
Eat less than you felt you should because there wasn't enough money for food	83.3 (5)
Hungry but didn't eat because you couldn't afford enough food.	83.3 (5)
	
**Overall food security status (scores 0 - 6)**	
High food insecurity (score = 6)	83.3 (5)
Food insecurity (score = 2)	16.7 (1)

^a ^Frequency that food did not last and didn't have money to get more

^b ^Frequency that could not afford to eat balanced meals

^c ^Frequency that cut the size or skipped meals because there wasn't enough money for food

Food-related activities that occurred prior to each of the five in-home assessments were documented. Overall, all participants purchased groceries prior to 3-4 of the 5 HFIs. The average total amount spent to purchase groceries across the 5 HFIs was $310.5 ± $124.04 (range $150 to $490). Almost all participants relied on someone else for transportation for most of their trips to purchase groceries. Fast food was not regularly purchased; however, when it was purchased, it was only once during a week. Full-service restaurants were not frequented by any of the participants.

The first household food inventory required 45 minutes to 1 hour to complete; the time required for the remaining four HFI s ranged from 30-45 minutes. Table 3 shows the number of households in which fresh and canned fruit and vegetables were present during the first household food inventory and the number of household inventories in which overall variety of fresh fruit and vegetables were present. Fresh fruit was not consistently present; five households had at least 2 different types of fruit, but only on one or two occasions. Bananas were the most frequently observed type of fresh fruit. However, the amount of bananas present ranged from 1-10 bananas and differed from week-to-week (data not shown). For example, one participant, with two adults and six children in the household, had bananas present during four HFI: 5 bananas at time 1, 1 at time 2, 0 at time 3, 1 at time 4, and 3 at time five (data not shown). Apples and oranges were never observed in any of the participant homes. Interestingly, if we used the first HFI (Time 1 column) as the only assessment of household availability of bananas, we would have missed the presence of bananas in four of the six households. Similarly, a single HFI would have identified half the households without any fresh fruit, instead of only one household that lacked fresh fruit on all five HFIs (data not shown). Carrots, lettuce, potatoes, and tomatoes were the most frequently observed fresh vegetables; the amount of each vegetable varied from week-to-week (data not shown). Reliance on the first HFI would have missed that no fresh vegetables were present in three of the six households on one occasion. Friedman's non-parametric two-way analysis of variation and Kendall's coefficient of concordance showed that there was no weekly agreement on the total number of fresh vegetables present (Kendall = 0.45, *p *= 0.04), the presence of any carrots (Kendall = 0.55. *p *= 0.016), and the amount of carrots (Kendall = 0.54, *p *= 0.019).

**Table 3:** Percentage and Number of Participant Mothers with Fresh and Canned Fruit and Vegetables Present During Five Household Food Inventories

		Number of Household Inventories in Which Foods Were Present					
	*Time 1* *% (n)*	*All 5* *% (n)*	*4 of 5* *% (n)*	*3 of 5* *% (n)*	*2 of 5* *% (n)*	*1 of 5* *% (n)*	*0 of 5* *% (n)*
***Fresh fruit - variety***^***a***^							
0	50.0 (3)	16.7 (1)	0	16.7 (1)	16.7 (1)	33.3 (2)	16.7 (1)
1	33.3 (2)		0	66.7 (4)	0	0	33.3 (2)
2	16.7 (1)	0	0	0	16.7 (1)	50.0 (3)	33.3 (2)
≥3	0	0	0	0	16.7 (1)	83.3 (5)	
***Fresh vegetables - variety***^***b***^							
0	0	0	0	0	0	50.0 (3)	50.0 (3)
1-2	16.7 (1)	0	0	16.7 (1)	0	16.7 (1)	66.7 (4)
≥3	83.3 (5)	50.0 (3)	16.7 (1)	16.7 (1)	0	16.7 (1)	0
***Canned fruit - variety***							
0	100 (6)	100 (6)	0	0	0	0	0
***Canned vegetables - variety***^***c***^							
0	0	0	0	0	16.7 (1)	0	83.3 (5)
1-2	16.7 (1)	0	0	50.0 (3)	0	33.3 (2)	16.7 (1)
≥3	83.3 (5)	16.7 (1)	33.3 (2)	0	33.3 (2)	0	16.7 (1)

^a ^Total number of different types of fresh fruit (0, 1, 2, and 3 or more): apples, avocados, bananas, guava, grapes, mango, melons, oranges, papaya, plantain, and strawberries.

^b ^Total number of different types of fresh vegetables (0, 1-2, and 3 or more): broccoli, cabbage, carrots, celery, corn, cucumber, greens, lettuce, peppers, potatoes, squash, and tomatoes.

^c ^Total number of different types of fresh vegetables (0, 1-2, and 3 or more): asparagus, carrots, corn, green beans, green peas, greens, hominy, mixed vegetables, potatoes, and tomatoes.

There was no canned fruit in any of the households at any time. On average, less than two different types of canned vegetables were observed in the households during the five HFI. The first HFI (Time1) failed to identify one household that did not have canned vegetables present on two occasions; and identified one of three households with one or two types of canned vegetables on three occasions. Corn, mixed vegetables, and tomatoes were the most frequently observed types of canned vegetables. There was no weekly agreement on the variety of canned vegetables present (Kendall = 0.51, *p *= 0.02) or presence (Kendall = 0.47, *p *= 0.03) or amount (Kendall = 0.69, *p *= 0.0008) of canned corn or mixed vegetables (Kendall = 0.53, *p *= 0.02; Kendall = 0.65, *p *= 0.006) observed in the homes. The amount of canned tomatoes differed from week-to-week (Kendall = 0.51, *p *= 0.02). Legumes were primarily available as dry or canned beans. Dry beans were present in all households on at least four of the five occasions. In addition, there was not agreement in the presence (Kendall = 0.43, *p *= 0.05) and amount (Kendall = 0.47, *p *= 0.03) of canned beans.

Dairy products are shown in Table 4. Low-fat milk, low-fat cheese, and low-fat ice cream were not consistently observed. Although not statistically significant, there was little agreement in weekly presence or amount of regular milk; however, lack of agreement across household observations of hard cheese was significant (Kendall = 0.44, *p *= 0.04). Dairy provides another example where a single observation (first HFI) differed from remaining HFIs. The first HFI identified half of the households (*n *= 3) that had whole milk or any milk on at least one occasion; low fat milk was not identified in any of the three households with low fat milk on 1-2 other occasions. During the first HFI, regular ice cream was observed in one of three households that subsequently had ice cream.

**Table 4:** Percentage and Number of Participant Mothers with Dairy Present During Five Household Food Inventories

		Number of Household Inventories in Which Foods Were Present					
	*Time 1* *% (n)*	*All 5* *% (n)*	*4 of 5* *% (n)*	*3 of 5* *% (n)*	*2 of 5* *% (n)*	*1 of 5* *% (n)*	*0 of 5* *% (n)*
***Milk***							
Whole	50.0 (3)	0	33.3 (2)	0	50.0 (3)	16.7 (1)	0
Low fat	0	0	0	0	16.7 (1)	33.3 (2)	50.0 (3)
Any milk	50.0 (3)	16.7 (1)	33.3 (2)	0	50.0 (3)	0	0
***Cottage cheese***							
Regular	16.7 (1)	0	0	0	0	33.3 (2)	66.7 (4)
***Yogurt***							
Regular	0	0	0	0	16.7 (1)	16.7 (1)	66.7 (4)
***Cheese***							
Regular	33.3 (2)	33.3 (2)	16.7 (1)	16.7 (1)	16.7 (1)	16.7 (1)	0
***Ice cream***							
Regular	16.7 (1)	0	0	0	33.3 (2)	16.7 (1)	50.0 (3)

Household availability of meats, poultry, seafood, and other protein foods are shown in Table 5. Regular beef was present during at least three inventories in five of the households; in one household, beef was not available during the first HFI, but was observed during the subsequent four inventories; and in another household, beef was observed during the first inventory, but not during the following two inventories. The first HFI failed to identify pork, hot dogs, chicken (breast, whole/pieces, or breaded), fish, and peanut butter in at least half of the households where they were present during other HFIs. The presence and amount of bacon was not consistent across the five inventories (Kendall = 0.43, *p = *0.05; Kendall = 0.46, *p *= 0.03). Although ham or bologna lunch meat was available at least once in five households, it was not present in any household during the first HFI. In half the households, chicken (whole/pieces) was not available during the first HFI, but was present on at least one occasion for all households; and in subsequent HFIs, the amount present varied from week-to-week (data not shown). Canned fish was available in all households on at least three occasions. In two of the households, canned fish was not observed during the first HFI; and amount of fish present was not consistent from week-to-week (Kendall = 0.53, *p *= 0.02). There was not agreement in the presence (Kendall = 0.56, *p *= 0.01) and amount (Kendall = 0.69, *p *= 0.0004) of peanut butter across the five HFIs. In two of the households where peanut butter was observed on at least two occasions, none was present during the first inventory.

**Table 5:** Percentage and Number of Participant Mothers with Meat/Poultry/Seafood and Other Protein Foods Present During Five Household Food Inventories

		Number of Household Inventories in Which Foods Were Present					
	*Time 1* *% (n)*	*All 5* *% (n)*	*4 of 5* *% (n)*	*3 of 5* *% (n)*	*2 of 5* *% (n)*	*1 of 5* *% (n)*	*0 of 5* *% (n)*
***Beef - regular***	66.7 (4)	33.3 (2)	33.3 (2)	16.7 (1)	0	0	16.7 (1)
***Pork***							
Regular	33.3 (2)	0	0	16.7 (1)	16.7 (1)	50.0 (3)	16.7 (1)
Sausage	0	0	0	0	16.7 (1)	33.3 (2)	50.0 (3)
Bacon	50.0 (3)	16.7 (1)	0	16.7 (1)	16.7 (1)	0	50.0 (3)
Chorizo	0	0	0	0	0	50.0 (3)	50.0 (3)
***Hot dogs***							
Beef/pork	0	0	0	0	0	16.7 (1)	83.3 (5)
Turkey/chicken	50.0 (3)	0	0	50.0 (3)	33.3 (2)	16.7 (1)	0
Corn dogs	0	0	0	0	0	16.7 (1)	83.3 (5)
***Lunch meat***							
Ham/bologna	0	0	16.7 (1)	0	50.0 (3)	16.7 (1)	16.7 (1)
Salami	0	0	0	0	50.0 (3)	0	50.0 (3)
Canned	0	0	0	0	0	33.3 (2)	66.7 (4)
***Chicken***							
Breast	16.7 (1)	0	0	0	16.7 (1)	33.3 (2)	50.0 (3)
Whole/pieces	50.0 (3)	16.7 (1)	16.7 (1)	16.7 (1)	33.3 (2)	16.7 (1)	0
Breaded	16.7 (1)	0	0	0	16.7 (1)	33.3 (2)	50.0 (3)
Canned	0	0	0	0	16.7 (1)	0	83.3 (5)
***Fish***							
Not breaded	0	0	0	0	16.7 (1)	16.7 (1)	66.7 (4)
Canned fish	66.7 (4)	66.7 (4)	0	33.3 (2)	0	0	0
***Peanut butter***							
Regular	33.3 (2)	33.3 (2)	16.7 (1)	0	16.7 (1)	0	33.3 (2)

Table 6 shows the availability of cereal, breads, crackers, prepared desserts, noodles, rice, and chips. The amount of unsweetened and sugar-sweetened cereal varied greatly from week-to-week; in at least two of the households, unsweetened and sugar-sweetened cereals were not available during the first HFI, but were available on at least two subsequent occasions. In the case of oatmeal, there was not agreement in the amount present across the inventories (Kendall = 0.48, *p *= 0.03). With the exception of one household, white bread was not available every week; and in three of the households, not on the first in-home assessment. Although present in two households on at least one occasion, whole wheat bread was not available during the first HFI. Corn tortillas were available on a regular basis in all households; however, the amount varied greatly from week-to-week (Kendall = 0.41, *p *= 0.06). In the five households where regular crackers were present on at least one occasion, none were observed during the first HFI. The same was true for all four households where regular cookies were observed during at least one HFI. There was lack of agreement in weekly presence (Kendall = 0.46, *p *= 0.03) and amount (Kendall = 0.62, *p *= 0.008) of pasta; and in the amount of white rice (Kendall = 0.38, *p *= 0.09).

**Table 6:** Percentage and Number of Participant Mothers with Cereals, Breads, Crackers, Prepared Desserts, Noodles, Rice, and Chips Present During Five Household Food Inventories

		Number of Household Inventories in Which Foods Were Present					
	*Time 1* *% (n)*	*All 5* *% (n)*	*4 of 5* *% (n)*	*3 of 5* *% (n)*	*2 of 5* *% (n)*	*1 of 5* *% (n)*	*0 of 5* *% (n)*
***Dry Cereal***							
Unsweetened	66.7 (4)	0	0	50.0 (3)	33.3 (2)	16.7 (1)	0
Sweetened	50.0 (3)	16.7 (1)	16.7 (1)	0	50.0 (3)	0	16.7 (1)
***Oatmeal***	66.7 (4)	16.7 (1)	66.7 (4)	0	0	16.7 (1)	0
***Maize atole***	0	0	0	16.7 (1)	0	0	0
***Bread***							
White	50.0 (3)	16.7 (1)	0	16.7 (1)	33.3 (2)	33.3 (2)	0
Whole wheat	0	0	0	16.7 (1)	0	16.7 (1)	66.7 (4)
***Tortillas***							
Corn	83.3 (5)	50.0 (3)	16.7 (1)	33.3 (2)	0	0	0
Flour	33.3 (2)	0	0	16.7 (1)	16.7 (1)	33.3 (2)	33.3 (2)
***Crackers***							
Regular	0	0	33.3 (2)	33.3 (2)	0	16.7 (1)	16.7 (1)
Low fat	0	0	0	16.7 (1)	0	0	83.3 (5)
***Prepared Desserts***							
Donuts	16.7 (1)	0	0	0	16.7 (1)	50.0 (3)	33.3 (2)
Regular cookies	0	0	0	33.3 (2)	16.7 (1)	16.7 (1)	33.3 (2)
***Noodles and Rice***							
Pasta	66.7 (4)	33.3 (2)	33.3 (2)	0	0	16.7 (1)	16.7 (1)
White rice	83.3 (5)	33.3 (2)	33.3 (2)	0	33.3 (2)	0	0
***Chips***							
Regular	0	0	16.7 (1)	0	0	16.7 (1)	66.7 (4)
Corn tostadas	16.7 (1)	0	16.7 (1)	0	0	50.0 (3)	33.3 (2)
*Chicharrones*	0	0	0	0	0	33.3 (2)	66.7 (4)

Regular chips were not present in any of the households during the first HFI; and in the household where regular chips were observed on four occasions, the amount varied greatly from week-to-week (data not shown). Although available on at least one occasion in 2 households, *chicharrones *(fried pork skins) were not observed during the first HFI. Interestingly, none of the households had nuts or candy.

Beverage availability is shown in Table 7. There was a lack of agreement on the amounts of regular soda across the assessments (Kendall = 0.47, *p *= 0.03). In five of the six households, sugar-sweetened soda was not observed during the first HFI, but during subsequent HFIs. The same was true for 100% fruit juice in four of five households and sugar-sweetened drink concentrate in five of the six households.

**Table 7:** Percentage and Number of Participant Mothers with Beverages Present During Five Household Food Inventories

		Number of Household Inventories in Which Foods Were Present					
	*Time 1* *% (n)*	*All 5* *% (n)*	*4 of 5* *% (n)*	*3 of 5* *% (n)*	*2 of 5* *% (n)*	*1 of 5* *% (n)*	*0 of 5* *% (n)*
***Soda***^a^	16.7 (1)	16.7 (1)	16.7 (1)	16.7 (1)	33.3 (2)	16.7 (1)	0
***Bottled water***	83.3 (5)	16.7 (1)	33.3 (2)	16.7 (1)	16.7 (1)	16.7 (1)	0
***100% fruit juice***	16.7 (1)	0	33.3 (2)	0	33.3 (2)	16.7 (1)	16.7 (1)
***Fruit drinks***	0	0	0	0	0	50.0 (3)	50.0 (3)
***Drink concentrate***^a^	16.7 (1)	16.7 (1)	0	33.3 (2)	16.7 (1)	33.3 (2)	0

^a ^Sugar-sweetened

**Figure 1:**
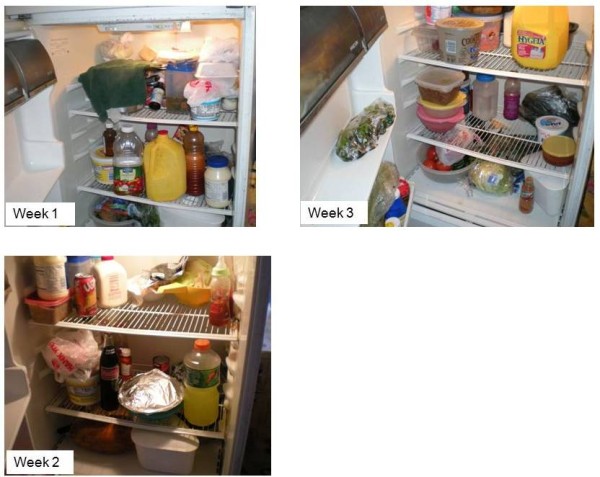
**Pictures taken by *promotoras *show the change in refrigerator food in family #1 during weeks 1, 2, and 3**.

**Figure 2:**
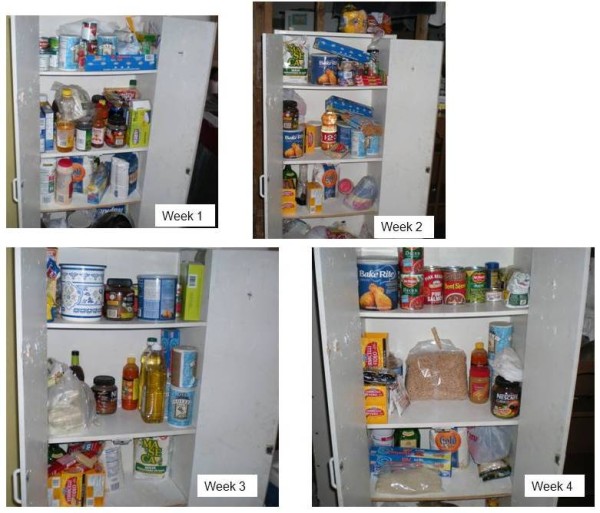
**Pictures taken by *promotoras *of pantry food contents in Family #3 during weeks 1, 2, 3, and 4**.

The photographs in figures 1 and 2 visually depict week-to-week change in refrigerator and cabinet contents in two of the participant households. Figure 1 shows pictures of the inside of the refrigerator taken in one family during the first three household food inventories. Whole milk was available during the first and third inventories; but not on the second, when low-fat milk was present for the only time during the month. In the same household, lettuce was available on during the first two inventories, not on the third. Figure 2 shows the inside of the same pantry in family #3 during the first four HFIs. The presence of dried beans, canned vegetables, and breakfast cereal was not consistent.

## Discussion

This study extends our understanding of the measurement of food availability in low-income *Mexicano *households that are located in Texas *colonias*, by 1) examining the feasibility of recruiting and retaining a sample of low-income households in a study that involved direct observation of the presence and amounts of a comprehensive list of food items in the home on five occasions over a thirty-day period; and 2) documenting the variation in household food supplies over time. Specifically, we describe the frequency that food items were present; examined weekly agreement in the presence and amount of food items; and compared a one-time measurement with multiple measurements. This is in contrast to the preponderance of household food inventory (HFI) studies in which availability was determined, whether through self-report or direct observation, at a single point in time [20,22,40,43,45,54,57,62,63]; rarely were details of the amount of food items examined [20,45,74]. In fact, little has been reported about the intra-month changes in household food supplies. Building on the work of Sisk and colleagues who conducted a similar project with nine African American and non-Hispanic White women in an urban area of approximately 72,000 people [65], our HFI instrument was modified to make it culturally and linguistically sensitive to *Mexicano *families in the *colonias *and to *promotora *researchers; and is apparently the first study to use trained *promotora *researchers to document household food availability (presence and amount) on five occasions among low-income households in areas of high neighborhood deprivation along the Texas border with Mexico [3]. Understanding the variation over time in availability of foods in the household is critical to informing decision-makers, formulating nutrition-related policy, and developing and implementing nutrition education programs that facilitate the consumption of healthy foods.

The results of this study indicated that it was feasible to recruit and retain a sample of low-income *Mexicano *households into a study that included five in-home assessments of a broad range of foods. Although a small sample, all six participant households in this feasibility study, which was conducted in two areas of *colonias *in the eastern and western parts of Hidalgo County, completed all surveys and allowed *promotora *researchers to complete direct observation and documentation of all food storage areas on five separate occasions. However, we must acknowledge the resource-intensity of this approach. In addition to the time necessary for scheduling in-home appointments and driving to each participant's home on five occasions, the household food inventory itself required the work of a two-person team of *promotoras *and 30-60 minutes for each data collection episode. As in other research projects in the *colonias*, the *promotoras *were the essential link to recruiting and retaining participant households [39], and to ensuring the cultural sensitivity of research that recognizes that data accuracy depends in part on how much the participants trust the researchers [75].

Overall, survey results indicated that there was a flow of food purchases over the month; all households purchased groceries, primarily at supermarkets or supercenters, on at least four occasions prior to the five in-home assessments of food availability. One of the factors that may influence the frequency of food purchases was the timing of household income; five of the six households received income on a daily or weekly basis. The multiple household food inventories (HFI) provided detailed and valuable information about the week-to-week change in the presence and amount of specific food items. Not only was there lack of agreement in the types of food items present from week-to-week, but also the amount. Bananas provided an excellent illustration of the importance of knowing the amount present. In one household of 8 individuals (2 adults and 6 children), bananas were present on four occasions; however, the amount present varied from 1-5 bananas. This suggests that although bananas were present, they were available to few within the household. Fresh vegetables showed a lack of agreement in weekly presence or amount of individual or total fresh or canned vegetables. Further, the presence and/or amount of dairy, meats (including poultry, seafood, and peanut butter), grains (cereals, breads, tortillas, cookies, pasta, and rice), and sugar-sweetened beverages was not consistent across the five household food inventories.

Further, the results revealed the inadequacy of a one-time measurement of household food resources, compared with multiple measures. If the first household food inventory was a one-time measure, it would have mistakenly identified at least one-half of the participant households without fresh fruit, canned vegetables (corn, mixed vegetables), dairy (whole or low-fat milk, cheese), protein foods (pork, hot dogs, chicken, fish, and peanut butter), grains (bread, crackers, and cookies), chips, and sugar-sweetened beverages (soda and fruit drinks). With some of the food items, none were observed during the first inventory, but present in multiple subsequent inventories. There were also food items that were present during the first inventory, but not present on subsequent inventories.

There are several major strengths to this study, especially in relation to other studies. Instead of using a single measure (on one occasion) of household food supplies, usually self-reported presence (and not quantity) of a limited number of food categories and items [54,55,57,58,62,76,77], this study extends our understanding of household food purchasing for in-home consumption among low-income households and illustrates the dynamic nature of the presence and amount of specific food and beverage items in the home. This was accomplished with five comprehensive in-home assessments, using the direct observation of the presence and amount of all food items by trained *promotora *researchers, and surveys that identified food-related activities within a week of each assessment and time since the last shopping trip. This is apparently the first study to examine household food availability among low-income *Mexicano *families in Texas *colonias *that are at high risk for nutrition-related health conditions and food insecurity. Food availability may be impacted by limitations in safe food storage and refrigeration, and by a number of cyclical and irregular events including family celebrations, bouts of acute illness and moments of familial stress By using five HFIs over a month, this study was better able to capture the impact of these events and to account for the flow of food purchases using dimensions of presence, amounts, and time window for intra-household variability.

There are several limitations that need to be addressed in future studies of household food availability. First, the small sample limited our ability to examine factors associated with presence and change in household food resources. The small sample may bias the feasibility of multiple HFIs; this will need to be evaluated in larger studies. Second, the results may not be generalizable beyond low-income, food insecure, *Mexicano *families. However, feasibility of multiple household food inventories and week-to-week variability in food resources was found in a pilot study of African American and non-Hispanic White women in an urban area [65]. Third, others have suggested that for a complete picture of household food purchasing behavior, eating out food purchases need to be documented [20]; however, the results of this study demonstrated these households rely little on fast food, full-service restaurants, or foods prepared outside the home and purchased for in-home consumption. Fourth, similar to other studies, there is no consideration given to seasonality. Future plans call for multiple HFI collected during each of the seasons: fall/winter, spring, and summer. There were challenges with accurately documenting the amount of a food item present (e.g., size of fruit and vegetables). In addition, the collection of multiple measures of household food resources by trained data collectors is resource intensive. Finally, future work will need to address the number and frequency of HFI necessary to describe "usual" household food availability. The appropriate number and time frame for household food inventories may depend on their purpose. A single HFI may correlate with a single 24-hour dietary recall; however, a single HFI may not correlate with multiple 24-hour dietary recalls or with screening questions that examine an individual's typical eating behavior.

## Conclusions

Access to healthy foods can play a pivotal role in the nutritional health of low-income *Mexicano *families in the expanding *colonias *of the Texas border with Mexico. Many of these families live in socioeconomically-deprived neighborhoods; many have a low household income, lack regular access to a vehicle, and reside a considerable distance from a supermarket [3]. Since the availability and accessibility of certain foods within the home has been strongly associated with food choice [58,78], documentation of the types and amounts of foods and beverages usually available in the home, whether targeting the prevention or management of a specific disease or condition or as a predictor of eating behavior in children, adolescents, or adults is critically important. In responding to methodological concerns in measuring household food availability [20], the findings from this study provide detailed information on the availability and amount of household foods and beverages by conducting multiple, direct observation household food inventories over a 30-day period. This study highlights the value of documenting weekly household food supplies, especially in households where income resources may be more volatile. Clearly, the data show that a single HFI may miss the changes in availability - presence and amount - that occur among low-income households that face challenges that require frequent purchase of foods and beverages. In targeting low-income and limited resource individuals and families, multiple household food inventories may be valuable for informing decision-makers, developing nutrition-related policy, and improving nutrition education. Researchers must be willing to take the steps necessary for rigorous measurement of a dynamic, household food environment. The measurement of household food availability must take into consideration frequency of income, home storage and refrigeration, and household number and composition, especially in underserved areas, where individuals are at increased risk for nutrition-related health conditions and household food supplies may not be consistent throughout the month [68,69]. This will require sufficient financial and people resources, and good relations with participants who allow strangers into their homes on multiple occasions.

## Competing interests

The authors declare that they have no competing interests.

## Authors' contributions

JRS developed the original idea for assessing household food availability. JRS worked with JASJ on the development of the instrument and the protocol for collection of data in the *colonias*. JRS wrote the first draft of the paper. JCH conducted the small sample statistical analysis. JRS, WRD, JASJ, and JCH read and approved the final manuscript.

## Pre-publication history

The pre-publication history for this paper can be accessed here:

http://www.biomedcentral.com/1471-2458/10/445/prepub
